# Psychosocial Consequences of Hand Eczema—A Prospective Cross-Sectional Study

**DOI:** 10.3390/jcm12175741

**Published:** 2023-09-03

**Authors:** Adam Zalewski, Piotr K. Krajewski, Jacek C. Szepietowski

**Affiliations:** Department of Dermatology, Venereology and Allergology, Wroclaw Medical University, 50-368 Wroclaw, Poland

**Keywords:** hand eczema, itch, pain, chronic inflammation, inflammatory disease, quality of life, anxiety, depression

## Abstract

Background: Hand eczema (HE) is a chronic inflammatory disease with a high prevalence, negatively influencing patients’ quality of life (QoL). It may also affect patients’ psychological status. The aim of this study was to assess and characterize the psychological burden of HE, its influence on patients’ QoL, and the presence and severity of anxiety and depressive disorders in HE patients. Methods: The study group consisted of 100 adult HE individuals. To assess the severity of the disease, two instruments were used: the Investigator Global Assessment for Chronic Hand Eczema (IGA-CHE) scale and the Hand Eczema Severity Index (HECSI). Assessment of patients’ quality of life (QoL) was obtained with the use of the DLQI tool. Patient Health Questionnaire-9 (PHQ-9) and Generalized Anxiety Disorder-7 (GAD-7) questionnaires were employed to assess depression and anxiety, respectively, as well as a modified version of the Hospital Anxiety and Depression Scale (HADS-M). Results: The mean DLQI value for the whole group reached 11.62 ± 6.35 points (13.27 ± 6.67 points in females and 9.15 ± 4.95 points in males; *p* = 0.023). A decrease in QoL correlated positively with the severity of the disease and the severity of itch and pain. In 17 patients (17%), a possible diagnosis of depressive disorder was found. Patients scoring higher results on the PHQ-9 and HADS-M depression (D) questionnaires reported greater intensity of the itch (r = 0.363, *p* < 0.001, and r = 0.237, *p* = 0.017, respectively) and the pain (r = 0.445, *p* < 0.001, and r = 0.287, *p* = 0.004, respectively). The anxiety disorder might possibly be diagnosed in 25% of patients (n = 25). This study revealed a positive correlation between the severity of the anxiety symptoms, measured with the use of both GAD-7 and HADS-M anxiety (A) tools, and the intensity of the pain (r = 0.248, *p* = 0.013, and r = 0.342, *p* = 0.001, respectively). The severity of depressive and anxiety symptoms correlated positively with the severity of the disease. Conclusions: The psychosocial burden of HE is an undeniable phenomenon. The disorder influences patients’ QoL and may cause mental disturbances such as depression and anxiety disorders.

## 1. Introduction

Hands can be defined as a terminal part of a human’s arm, responsible for touching, grabbing, moving, or feeling things. Thanks to their mobility and visibility, they play an important role in not only work or social life but also verbal (writing or sign language) and non-verbal communication. Hand eczema (HE), being a chronic inflammatory disease of high prevalence, depends on various etiological factors [1]. The clinical picture of HE is heterogeneous, and the course of the disease presents a wide spectrum. Itching and pain are described as two of the most burdensome symptoms of HE, correlating with the severity of the disease [2,3]. Also, because of the localization of skin lesions on hands and often challenging treatment, the disorder frequently places a major psychological burden on patients suffering from HE [1,4]. It undeniably negatively influences interpersonal relations and quality of life (QoL), causing embarrassment, stigmatization, and social withdrawal [5,6]. According to data available in the literature, the disease also has a major financial consequence, causing days lost through illness and the need to use sick leave because of the hands’ condition and associated symptoms [5,7]. As a result of all the above-mentioned factors, HE may severely affect patients’ psychological status, causing depressive or anxiety disorders, a reduction in self-esteem, or sexual dysfunctions [5,8]. 

In this study, the authors aimed to assess and characterize the psychosocial burden of HE, the influence of the disease on QoL, and both the presence and severity of anxiety and depressive disorders in HE patients. 

## 2. Materials and Methods

### 2.1. Studied Group

A cross-sectional, prospective study was performed. The studied population comprised a group of 100 consecutive individuals. All participants were patients of the Department of Dermatology, Venereology, and Allergology in Wroclaw, Poland, where they were either admitted as inpatients to the hospital ward or received treatment as outpatients at the outpatient clinic. The authors of the study (dermatology specialists and dermatology residents) recruited them on the day of admission. A total of 60% of the group were women (n = 60) and 40% were men (n = 40). Population age ranged from 18 to 80 years (mean 46.0 ± 17.23). The diagnosis of HE was made based on clinical manifestation criteria. The mean duration of the disease was determined at 42.5 ± 60.84 months and ranged from 3 to 396 months. Study inclusion criteria were as follows: ≥18 years old (adult age) and a course of the disease lasting over 3 months (chronic HE diagnosis). All subjects with suspicion of CHE but waiting for the final diagnosis to be made were excluded from the study (uncertain biopsy result, unclear clinical picture). Detailed group characteristics may be found in Table 1. 

This study was approved by the local ethics committee (Consent No. KB-234/2023), and written informed consent was obtained from all studied individuals.

### 2.2. Disease Severity Assessment

To assess the severity of the disease, two instruments were used: the Investigator Global Assessment for Chronic Hand Eczema (IGA-CHE) scale [9] and the Hand Eczema Severity Index (HECSI) [10]. 

IGA-CHE (Investigators Global Assessment; IGA) classifies the severity of HE into five categories: ‘clear’ (IGA-CHE 0), ‘almost clear’ (IGA-CHE 1), ‘mild’ (IGA-CHE 2), ‘moderate’ (IGA-CHE 3), and ‘severe’ (IGA-CHE 4) [9]. 

Another approach employed for assessing the disease severity in patients with HE included using the HECSI scale. This scale incorporates the intensity, extent, and clinical manifestations of the ailment. The hands of each patient are divided into five distinct regions: fingertips, fingers (excluding the fingertips), palm of the hand, back of the hand, and wrists. Within each of these regions, the intensity of six specific clinical indicators—erythema, induration/papulation, vesicles, fissuring, scaling, and edema—is assessed and graded using the following scale: 0 (indicating no observable skin changes), 1 (indicating mild disease), 2 (indicating moderate disease), and 3 (indicating severe disease). For each individual area, the cumulative affected area is scored between 0 and 4 to indicate the extent of clinical symptoms (0 = 0%, 1 = 1–25%, 2 = 26–50%, 3 = 51–75%, and 4 = 76–100%). The score assigned to the extent of clinical symptoms within each area is then multiplied by the total sum of the intensity levels for each clinical feature and added together. The final HECSI score ranges from 0 to 360 points, with 360 representing the highest level of severity [10]. To classify patients into severity groups, the following cutoff values were applied: clear (0 points; HECSI 0), almost clear (1–16 points; HECSI 1), moderate (17–37 points; HECSI 2), severe (38–116 points; HECSI 3), and very severe (117 points or higher; HECSI 4) [10,11].

### 2.3. Itch and Pain Assessment

The intensity of itch and pain was assessed using the Numeric Rating Scale (NRS). Study participants were tasked with rating the intensity of the most severe itch and pain experienced in the three days preceding the study, as well as the most intense itch and pain since the disease’s onset. 

The NRS is a tool designed to evaluate the intensity of specific symptoms like itch or pain. This scale is unidimensional and spans from 0 (indicating no itch/pain) to 10 (representing the most intense itch/pain imaginable). The interpretation of the Itch-NRS scoring is as follows: no itch (0 points), mild itch (1–3 points), moderate itch (4–6 points), severe itch (7–8 points), and very severe itch (≥9 points) [12]. The following cutoffs on the pain-related NRS were implemented for pain assessment: ≤5—mild pain; 5–7—moderate pain; and 7–10—indicating severe pain on the 0–10 rating scale [13]. 

### 2.4. QoL Assessment

For the evaluation of patients’ quality of life (QoL), the Polish version of the Dermatology Life Quality Index (DLQI) questionnaire was employed [14]. The DLQI is a specialized dermatological tool designed to assess symptoms and emotions, daily activities, leisure, work and school-related aspects, relationships, and treatment side effects over the preceding 7 days. It is composed of 10 items, each assigned a score ranging from 0 to 3 points (0 indicating ‘not at all’; 1 indicating ‘a little’; 2 indicating ‘a lot’; 3 indicating ‘very much’). These scores are then summed to obtain a total DLQI score, which ranges from 0 to 30 points. A score of 0–1 point signifies the minimal impact of the disease on QoL; 2–5 points indicate a small impact; 6–10 points represent a moderate impact; 11–20 points suggest a large impact; and 21–30 points indicate an extremely large impact [14,15,16].

### 2.5. Depression and Anxiety Assessment

Various questionnaires (Polish versions) were used to screen for depression and anxiety among the studied population, such as the Patient Health Questionnaire-9 (PHQ-9) [17], the 7-item anxiety scale (GAD-7) [18], and the Hospital Anxiety and Depression Scale (HADS-M) [19].

The PHQ-9 is a nine-item tool created to screen for depression in different medical settings. Patients are assessing the incidence of each of nine major depressive disorder diagnosis criteria (based on the Diagnostic and Statistical Manual of Mental Disorders, DSM-IV) occurrences, scoring 0 (‘not at all’), 1 (‘several days’), 2 (‘more than half of the days’), and 3 (‘nearly every day’). Standard cutoff scores were used as 5, 10, 15, and 20, representing cutoff points for mild, moderate, moderately severe, and severe depression, respectively. When screening for a depression diagnosis, a result of 10 points or greater presents 88% of both sensitivity and specificity for the possibility of a major depression diagnosis and is defined as a diagnostic cut-point [17,20,21]. 

The GAD-7 scale is a self-reported measure screening for the presence of generalized anxiety disorder (GAD) and the level of anxiety and is composed of seven items. Each item is a statement describing general somatic tension or worry and is rated on a 4-point Likert-type scale assessing symptom frequency in the range from 0 (‘not at all sure’) to 3 (‘nearly every day’). The higher the score, the higher the level of GAD symptoms. The sum of 5, 10, and 15 points were implemented as the cutoff values for mild, moderate, and severe anxiety, respectively. A score of 8 or higher is a cut-point for identifying the probable occurrence of generalized anxiety disorder [18,22,23,24].

HADS-M stands for a modified version of the Hospital Anxiety and Depression Scale (HADS) created by Zigmond et al. [19]. This variant comprises 16 queries, with a potential score of 0 to 3 points for each question. The highest achievable scores are distinct for depression (21 points), anxiety (21 points), and aggression (6 points). The scoring criteria adopted for the anxiety and depression subsections are as follows: no disorders (0–7 points), borderline states (8–10 points), and disorders (11–21 points) [25,26].

### 2.6. Statistical Analysis

The statistical analysis was performed using IBM SPSS Statistics v. 26 (SPSS Inc., Chicago, IL, USA) software. In the beginning, the normality of all data was assessed with the Shapiro–Wilk normality test. Subsequently, minimum, maximum, means, standard deviations, medians, and quartiles were calculated. In order to assess quantitative variables, the Student’s *t*-test and Mann–Whitney U test, depending on normality, were implemented. For qualitative data, the Chi2 test was used. For the assessment of differences between more than two groups, ANOVA or Kruskal–Wallis one-way analysis of variance on ranks was used. Post-hoc analysis with Bonferroni corrections was implemented for both tests. The two-sided *p*-value of less than 0.05 was considered statistically significant.

## 3. Results

### 3.1. Disease Severity Assessment 

Out of the examined population of 100 individuals (n = 100), the distribution across IGA-CHE categories was: group IGA-CHE 1 (almost clear) comprised 15.0% (n = 15), group IGA-CHE 2 (mild) included 25.0% (n = 25), group IGA-CHE 3 (moderate) accounted for 37.0% (n = 37), and group IGA-CHE 4 (severe) constituted 23.0% (n = 23). In terms of gender breakdown, the majority of men fell into IGA 1 and 2 groups (n = 11; 27.5% in both groups), while among women, IGA 3 was the predominant category (n = 28; 46.7%). Regarding the HECSI score, the mean value was 35.0 ± 27.8 points (29.3 ± 26.7 points in males and 38.8 ± 28.1 points in females).

### 3.2. QoL Assessment

The mean DLQI value for the whole group was assessed at 11.62 ± 6.35 points. In most cases, HE had a moderate (33%; n = 33) or very large (39%; n = 39) effect on patients’ QoL. In 18% of respondents, the disease’s effect on QoL was none or small (2%, n = 2, and 16%, n = 16, respectively), and 10 patients assessed that effect as extremely large. Considering the gender division among females, the mean DLQI value reached 13.27 ± 6.67 points, while the mean DLQI score for males amounted to 9.15 ± 4.95 points; the difference was statistically significant (*p* = 0.023). In 28 (46.7%) females, the influence on QoL was found to be very large, which was the most common result, whereas the most frequent in males was a moderate effect (n = 17; 42.5%). Detailed data are presented in Table 2. 

**Figure 1:**
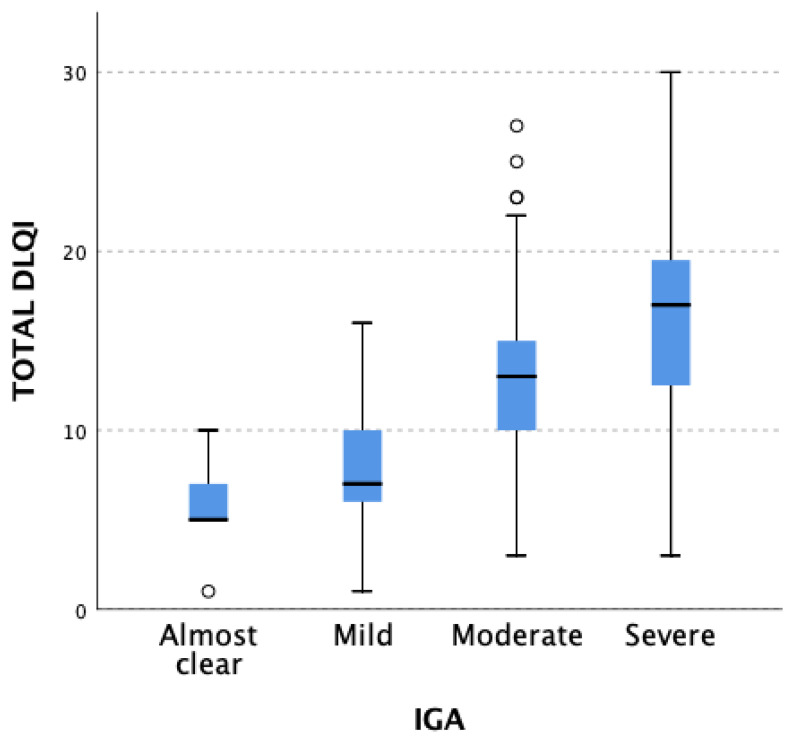
Differences in total DLQI score result in patients from particular IGA-CHE severity groups (*p* < 0.001). The statistically significant difference was observed when comparing the following IGA-CHE groups: 1 (almost clear) vs. 3 (moderate) (*p* < 0.001), 1 (almost clear) vs. 4 (severe) (*p* < 0.001), 2 (mild) vs. 3 (moderate) (*p* = 0.009), and 2 (mild) vs. 4 (severe) (*p* < 0.001). Mild–severity groups present a lower decrease in QoL. White circles correspond to patients with DLQI scores out of range.

Considering the whole studied population, statistically significant differences in QoL were found when comparing different IGA-CHE severity groups (*p* < 0.001). Post hoc analysis revealed that when comparing individual IGA-CHE severity groups, the difference in the decrease in QoL was statistically significant in four cases: 1 (almost clear) vs. 3 (moderate) (*p* < 0.001); 1 (almost clear) vs. 4 (severe) (*p* < 0.001); 2 (mild) vs. 3 (moderate) (*p* = 0.009); 2 (mild) vs. 4 (severe) (*p* < 0.001). In low disease severity groups, a lower decrease in patients’ QoL was observed. Outcomes are presented in Figure 1. 

**Figure 2:**
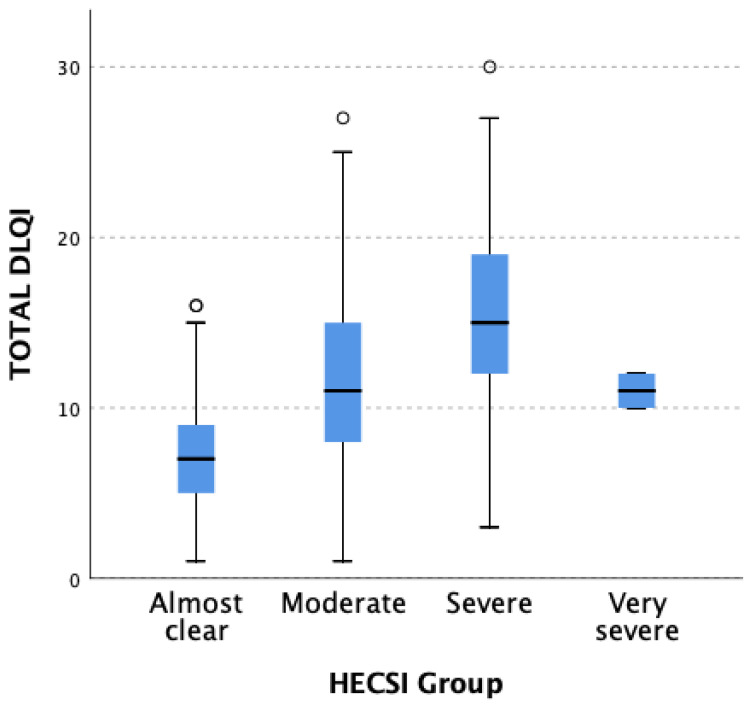
Differences in total DLQI score result in patients from particular HESCI groups (*p* < 0.001). The statistically significant difference was observed when comparing the following HECSI groups: 1 (almost clear) vs. 2 (moderate) (*p* = 0.023) and 1 (almost clear) vs. 3 (severe) (*p* < 0.001). White circles correspond to patients with DLQI scores out of range.

Similar observations were made concerning patients from different HECSI groups (*p* < 0.001). Statistically significant differences in reduction in QoL measured in DLQI were also found when comparing patients from the HECSI 1 (almost clear) group vs. the HECSI 2 (moderate) group (*p* = 0.023) and the HECSI 1 (almost clear) group vs. the HECSI 3 (severe) group (*p* < 0.001). Results are presented in Figure 2. 

A decrease in QoL correlated positively with the severity of the disease measured in IGA-CHE (r = 0.617; *p* < 0.001) and in HECSI (r = 0.579; *p* < 0.001).

The total DLQI score correlated positively with the severity of both assessed symptoms, itch and pain, in the 3 days prior to the study period (r = 0.436, *p* < 0.001, and r = 0.305, *p* = 0.002, respectively). No correlation between the DLQI score and the duration of the disease was found (*p* > 0.05). 

### 3.3. Depression Assessment (PHQ-9 and HADS-M (D)) 

#### 3.3.1. PHQ-9 

Based on the PHQ-9 cut-point score (≥10 points), among the whole studied population, in 17 patients (17%), a possible diagnosis of depressive disorder was documented. It was more common among females (n = 13; 21.7%) than males (n = 4, 10%), yet the difference between both groups was statistically insignificant. 

In relation to the whole group, the mean PHQ-9 score was 6.3 ± 4.9 points. The mean value in females was 7.12 ± 5.14 points and 5.08 ± 4.14 points in males. The difference was statistically insignificant. Table 3 demonstrates the distribution of patients in depression severity groups according to the PHQ-9 score. 

**Figure 3:**
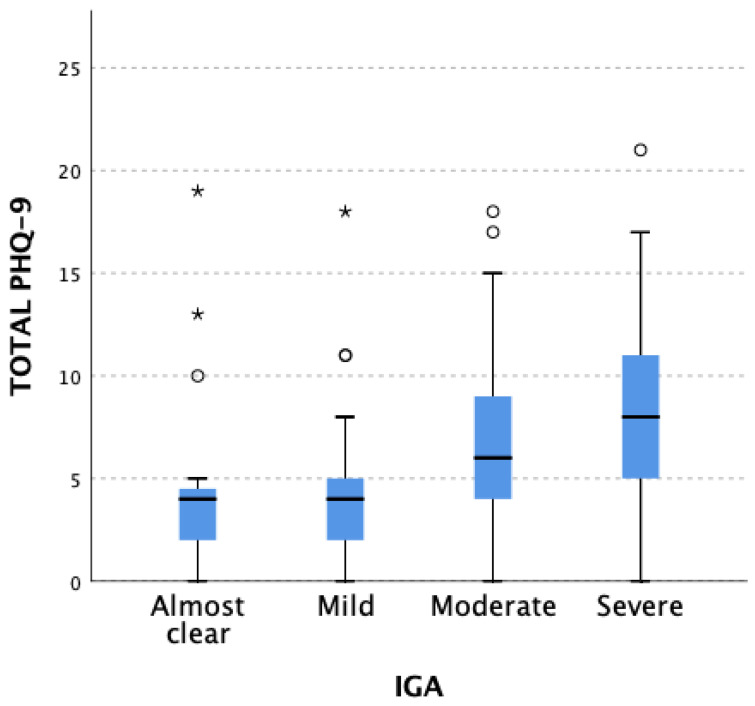
Differences in total PHQ-9 scores result in patients from particular IGA-CHE severity groups. Patients representing the IGA-CHE 4 (severe) group compared with the IGA-CHE 2 (mild) group showed significantly higher results of PHQ-9 (*p* = 0.028). When comparing other groups (1 (almost clear) vs. 2 (mild), 1 (almost clear) vs. 3 (moderate), and 3 (moderate) vs. 4 (severe)), results were numerically higher but did not achieve statistical significance. White circles and asterisks correspond to patients with PHQ-9 scores out of range.

Differences in total PHQ-9 score results in patients from particular IGA-CHE severity groups were found and are presented in Figure 3. When comparing IGA-CHE 4 (severe) group patients with IGA-CHE 2 (mild) group patients, significantly higher results of PHQ-9 (*p* = 0.028) were observed. In other IGA-CHE groups (1 (almost clear) vs. 2 (mild), 1 (almost clear) vs. 3 (moderate), and 3 (moderate) vs. 4 (severe)), results were numerically higher but did not achieve statistical significance. 

The correlation between the intensity of depressive symptoms and the severity of HE was detected for both HECSI (r = 0.264; *p* = 0.008) and IGA-CHE scores (r = 0.329; *p* = 0.001). Patients scoring higher on the PHQ-9 questionnaire reported greater intensity of the itch (r = 0.363; *p* < 0.001) and pain (r = 0.445; *p* < 0.001) in the last 3 days prior to the study. PHQ-9 scores also correlated with the decrease in QoL (r = 0.537; *p* < 0.001). Moreover, a positive correlation was found between PHQ-9 scores and other scales assessing not only depression (HADS-D: r = 0.664; *p* < 0.001) but also anxiety: GAD-7 (r = 0.617; *p* < 0.001) and HADS-A (r = 0.690; *p* < 0.001).

#### 3.3.2. HADS-M: Depression (D)

The distribution of patients in depressive disorder severity groups, considering gender division, is presented in Table 4.

For the whole group, the mean value of HADS-M (D) was 4.7 ± 3.1 points. Among females, it was assessed at 5.22 ± 3.29 points, whereas in males, it amounted to 3.83 ± 2.74 points. The difference was statistically significant (*p* = 0.029). 

The intensity of depressive symptoms measured in HADS-M correlated positively with the severity of the disease (for IGA-CHE: r = 0.283; *p* = 0.004, and HESCI: r = 0.228; *p* = 0.004, respectively), as well as with the intensity of the itch (r = 0.237; *p* = 0.017) and the pain (r = 0.287; *p* = 0.004). No correlation with the duration of the disease was found.

### 3.4. Anxiety Assessment (GAD-7 and HADS-M (A)) 

#### 3.4.1. GAD-7

In accordance with GAD-7 anxiety diagnostic criteria (a cut-point of 8 points or higher), anxiety disorder might be diagnosed in 25% of the whole group (n = 25): 17 females (28.3%) and 8 males (20%). The difference did not reach statistical significance.

The mean GAD-7 score for the whole studied population was assessed at 5.8 ± 4.0 points. It reached 6.17 ± 4.13 points in females and 5.23 ± 3.75 points in males, with no significant difference between sexes. Detailed data concerning GAD-7-score-dependent anxiety severity groups are shown in Table 5. 

Interestingly, an association between the severity of pain and the presence of an anxiety diagnosis was observed. The mean pain severity in patients with diagnosed anxiety was 3.48 ± 3.31 points, whereas the mean value in patients without anxiety amounted to 2.24 ± 2.93 points, both measured on the NRS scale. The difference was statistically significant (*p* = 0.034). No such dependency was observed for the itch (*p* > 0.05). 

The severity of the anxiety disorder diagnosis among the studied population correlated positively with the severity of the disease measured in IGA-CHE (r = 0.223; *p* = 0.026). There was no such correlation found for the HECSI score. The intensity of anxiety symptoms also correlated with the intensity of the pain (r = 0.248; *p* = 0.013). No such relationship was documented between anxiety scores and itch intensity (*p* > 0.05). No correlation between the severity of the anxiety and the duration of the disease was observed (*p* > 0.05). GAD-7 outcomes also correlated with results obtained with the HADS-M (A) questionnaire (r = 0.712; *p* < 0.001). 

#### 3.4.2. HADS-M: Anxiety (A)

The mean value of HADS-M (A) was 5.3 ± 3.0 points when considering the whole research population. The mean result of the evaluation in females was 5.87 ± 3.36 points and 4.40 ± 2.12 points in males. The difference was statistically significant (*p* = 0.001). Table 6 shows detailed data concerning the severity of anxiety disorders.

A positive correlation was observed between the intensity of anxiety symptoms and the severity of the disease, but solely for the IGA-CHE score (r = 0.230; *p* = 0.022). Similarly to the GAD-7 assessment, the results of HADS-M (A) correlated with the intensity of pain (r = 0.342; *p* = 0.001). However, no such association was found for the itch. Intriguingly, the intensity of anxiety symptoms exhibited a negative correlation with the disease’s duration (r = −0.215; *p* = 0.032). 

## 4. Discussion

The impact of HE on patients’ QoL is significant. In the paper published by Cazzaniga et al. [7] concerning 199 individuals suffering from HE, most patients reported a moderate (33.7%) or large (39.4%) effect of the disease on their QoL. The mean DLQI score amounted to 9.7 ± 5.8 points [7]. The impact of HE on DLQI was significantly higher among females, which was also observed in other studies [27,28]. The results of our research stay in line with these findings. Our study shows additionally that the severity of HE among women is higher, which can potentially result in a higher QoL decline. Mollerup et al. (2014) [29] created an analysis of gender differences in patients with hand eczema. They concluded that the higher decrease in QoL in females may be associated with the severity of the disease and the quantity of exacerbating factors (such as contact with detergents, hygiene products, handling of food, or handwashing). Also, a significant role of work-related and everyday exposures or routines in treatment and prevention was underlined [29]. Visual and practical aspects associated with the need to apply protection measures and the presence of skin lesions were underlined [7]. Additionally, the authors distinguished factors having a strong impact on DLQI, such as lesions localized on the back of the hands, the presence of the itch, or the necessity to wear gloves. Moreover, HE influenced patients’ ability to work: 37% of participants used sick leave, and 15% of them left or changed jobs because of the disease [7]. Data were comparable to the results of different studies [30]. 

More importantly, the process of sickness and being sick, as well as all their consequences, generate a cost for patients and the whole healthcare system. Loss of productivity, followed by hospitalization and travel expenses, were recognized as the most cost-consuming contributors [31]. 

Our study shows that several HE symptoms (itch, pain) may also be considered factors whose intensity interferes with the impairment of QoL. A correlation between itch severity and the increase in DLQI scoring was also demonstrated by Wang et al. [32]. On the contrary, in the study of Ruppert et al. [33], itch in severe and very severe forms of HE was observed to be correlated with small or moderate impairments in QoL. More studies in this area need to be carried out.

Apart from the severity of the itch, a positive correlation between pain severity and loss of QoL was observed among the group of our patients. Torisu-Itakura et al. [34], in the study concerning the impact of itch and skin pain on QoL in adult patients with atopic dermatitis in Japan, revealed that the coexistence of itch and skin pain may cause sleep disturbances or may impair work-related activities. Moreover, patients experiencing both itch and pain were more likely to complain and be bothered by their symptoms (*p* = 0.034). That same group of patients was more likely to be dissatisfied with the lack of improvement and inconveniences of the treatment [34]. Moberg et al. [35] also noticed that pain may be a symptom impairing HE patients’ QoL. In a population of young men and women (18–34 years), pain was described as a factor significantly influencing QoL [35]. 

Multiple studies show anatomical, physiological, functional, and ontogenetic connections between the skin and the nervous system. Various dermatoses have a major impact on patients’ psychological status, which may be confirmed by the comorbidity of certain chronic inflammatory skin diseases (such as psoriasis, atopic dermatitis (AD), or hidradenitis suppurativa (HS)) and several mental symptoms or syndromes. It has been estimated that in over 30% of patients suffering from dermatological disorders, psychiatric comorbidity is diagnosed [36]. Markers of inflammation were reported to be elevated in both skin and mental diseases [34,35,37,38]. 

The idea of an active inflammatory process taking place in the human system is becoming more and more significant among biological theories explaining probable causes of depression. Inflammation indicators that are considered to lead to a deficit in serotonin and melatonin (one of the major reasons for depressive disorder) are as follows: inflammatory enzymes (manganese superoxide dismutase (MnSOD), myeloperoxidase (MPO)), pro- and anti-inflammatory cytokines, and oxidative stress [39]. In some populations, a dysregulated immunological response has been linked to the onset of depression [40]. 

Systemic inflammation seems to be mainly correlated with depressive symptoms. However, anxiety disorders are also considered to be linked with increased inflammation, primarily because of the activation of the stress response and stimulation of immunological cells to release cytokines [41,42]. 

Patients suffering from chronic dermatoses such as HS are considered to be more likely to develop depressive symptoms. Studies available in the literature also suggest an association between HS and an elevated risk of anxiety [43,44]. The research of Rymaszewska et al. [37] seems to confirm these findings, showing a high prevalence of mental disorders among that particular group of patients. 

Also, up to 90% of patients with psoriasis were reported to have psychological comorbidity [45], with the predominance of anxiety disorders [46,47]. Exacerbation of psoriatic lesions is associated with increased production of inflammatory mediators, which may contribute to neurotransmitter imbalance and cause or intensify existing symptoms of depression and anxiety [48]. Despite the high prevalence of mood disturbances among patients with psoriasis, the common lack or delay of their diagnosis is underlined by some experts, potentially resulting in clinical consequences [49]. 

A systemic review by Rønnstad et al. [50] demonstrated an increased risk of the coexistence of AD with depression and anxiety in adults. Another study found that 20.1% of AD patients were diagnosed with depressive disorder, compared with 14.8% in the non-AD control group [51]. The pathophysiological and clinical importance of the IL-4/IL-13 axis in AD has been proven [52]. A reduction in depressive and anxiety symptoms (measured with HADS) was presented in two randomized placebo-controlled studies on dupilumab (a monoclonal antibody binding to IL-4/IL-13 receptors), which may link inflammation and mood disorders in the AD patient population [53,54]. 

In a 15-year follow-up study on HE, as many as 96% of patients reported that the disease influenced their psychosocial functions [55]. In 2018, Marron et al. [6] constructed a large European multicenter study to identify the psychological, social, and clinical characteristics of patients with HE. Statistically significant differences were found between female patients and female controls regarding anxiety and depression measured with HADS—symptoms of both disorders were more severe in HE individuals. Also, the severity of both anxiety and depression differed when comparing females and males (7.00 vs. 5.00 for anxiety, respectively; 4.00 vs. 3.00 for depression, respectively; both measured with HADS). A similar dependency was also observed in our study. Importantly, based on their results, a relationship between high suicidal ideation, low socioeconomic status, being widowed or divorced, and the possibility of an anxiety diagnosis was found (OR > 1; *p* = 0.038, *p* < 0.001, and *p* < 0.001, respectively). A comparable association was detected for low socioeconomic status (*p* = 0.007), being widowed or divorced (*p* = 0.001), and the potential diagnosis of a depressive disorder. Moreover, the threat of losing their job in patients with severe occupational hand eczema increased levels of anxiety and depression [56]. 

HE is also described as causing embarrassment and a loss of self-consciousness [5,57]. A study investigating the physical and aesthetic effects of HE conducted on a group of over 1000 participants revealed that 74% of the studied group reported the disease influencing the way they grab objects or touch people. In 70% of cases, patients admitted to wearing gloves or hiding their hands in their pockets because they were ashamed of their skin condition. HE also impacted relationships with partners, families, and friends. The disease kept study participants from taking part in some particular everyday situations [5]. 

Among others, obsessive–compulsive tendencies appeared to be noticed in HE patients. Kouris et al. [58] linked those behaviors with high anxiety levels. In their research, a significant difference in the Leyton Trait Scale (LTS, an instrument assessing obsessive–compulsive personality traits) was found between HE individuals and healthy controls. Furthermore, a positive correlation was observed between LTS, DLQI, HADS-A, and age. They implied that HE skin lesions might be self-induced in some cases, being triggered by compulsive actions or tendencies such as compulsive hand washing, scratching, rubbing, or skin picking. Yet, the exact cause-and-effect sequence is difficult to estimate [58]. 

An individual’s reaction to stress can be associated with hand dermatoses’ coping mechanisms. A study by Niemeier et al. [59] concerning different hand dermatoses (e.g., HE) distinguished a specific subgroup of patients who coped worse with their disease. High-stress responders (patients who identified stress as a factor influencing the disease) with negative results of patch tests were recognized as having a greater need for psychosocial therapy. It was explained by the disappointment caused by negative test results without clarifying the causes of the disease. Not only a psychological consultation but also long-term psychotherapy need to be considered in such cases [59].

Our study has some limitations worth mentioning. Conclusions drawn in our research, especially those related to depressive and anxiety disorder diagnoses, should be confirmed and followed by a detailed psychiatric examination and expertise. Nevertheless, our group proved the urgency of screening HE patients in terms of their mental status using simple, easily accessible screening tools. It would clearly help to distinguish patients belonging to high-risk groups for mental disorders. This study was also conducted on a limited and geographically undifferentiated population (all 100 participants were recruited from the Lower Silesia region in Poland). Future studies incorporating patients from other regions need to be considered. Although some correlations between disease severity, itch and pain intensity, and depressive and anxiety symptoms were found, the exact cause of mood disorders in HE patients remains unclear.

## 5. Conclusions

This study proves the psychosocial burden of HE. It emphasizes the substantial role of a multidisciplinary approach for patients suffering from HE. Understanding the way the disease affects patients’ lives may provide useful advice on treatment regimens or skincare, with adequate compliance. Exploring this part of the knowledge may help medical practitioners improve their management and early suspicion or diagnosis of depressive and anxiety disorders. Finally, such awareness could contribute to the prevention of mood disturbances and all their possible repercussions in HE patients. 

## Figures and Tables

**Table 1:** Group characteristics.

Characteristics	Whole Population (n = 100)	Females (n = 60)	Males (n = 40)	*p*
Age, years (mean ± SD)	46.0 ± 17.23	46.6 ± 18.27	36.9 ± 13.2	NS
Disease duration, months (mean ± SD)	42.5 ± 60.84	30.85 ± 40.34	27.7 ± 7.1	NS
Previous treatment	71 (71.0%)	47 (78.3%)	24 (60.0%)	0.048
Systemic treatment	28 (28.0%)	17 (28.3%)	11 (27.5%)	NS
History of atopy/allergy	45 (45.0%)	26 (43.3%)	19 (47.5%)	NS
Diagnosed allergic contact background	14 (14.0%)	8 (13.3%)	6 (15.0%)	NS
Previous patch testing	27 (27.0%)	13 (21.7%)	14 (35.0%)	NS
Itch in last 3 days	81 (81.0%)	28 (70.0%)	53 (88.3%)	0.022
Pain in last 3 days	53 (53.0%)	16 (40.0%)	37 (61.7%)	0.033
Lesion location				
Only hands	65 (65.0%)	38 (63.3%)	27 (67.5%)	NS
Hands and feet	23 (23.0%)	15 (25.0%)	8 (20.0%)	NS
Disseminated lesions	12 (12.0%)	7 (11.7%)	(12.5%)	NS

NS—‘not significant’.

**Table 2:** Distribution of patients in DLQI groups, considering gender division.

DLQI Group (Effect on Patients QoL)	Total Group Size/ Frequency (n = 100)	Females (n = 60)	Males (n = 40)	*p*
no effect	2 (2.0%)	1 (1.7%)	1 (2.5%)	0.023
small	16 (16.0%)	6 (10.0%)	10 (25.0%)	
moderate	33 (33.0%)	16 (26.7%)	17 (42.5%)	
very large	39 (39.0%)	28 (46.7%)	11 (27.5%)	
extremely large	10 (10.0%)	9 (15.0%)	1 (2.5%)	

**Table 3:** Distribution of patients in depression severity groups according to PHQ-9 score, considering gender division.

Depression Severity Group (According to PHQ-9)	Total Group Size/ Frequency (n = 100)	Females (n = 60)	Males (n = 40)	*p*
mild	43 (43.0%)	24 (40.0%)	19 (47.5%)	NS
moderate	35 (35.0%)	19 (31.7%)	16 (40.0%)	
moderately severe	13 (13.0%)	10 (16.7%)	3 (7.5%)	
severe	9 (9.0%)	7 (11.7%)	2 (5.0%)	

NS—‘not significant’.

**Table 4:** Distribution of patients in depressive disorder severity groups (according to HADS-M), considering gender division.

Depressive Disorder Severity Group (According to HADS-M)	Total Group Size/ Frequency (n = 100)	Females (n = 60)	Males (n = 40)	*p*
no disorders	80 (80.0%)	44 (73.3%)	36 (90.0%)	NS
borderline states	14 (14.0%)	11 (18.3%)	3 (7.5%)	
disorders	6 (6.0%)	5 (8.3%)	1 (2.5%)	

NS—‘not significant’.

**Table 5:** Distribution of patients in anxiety disorder severity groups (according to GAD-7), considering gender division.

Anxiety Severity Group (According to GAD-7)	Total Group Size/ Frequency (n = 100)	Females (n = 60)	Males (n = 40)	*p*
mild	43 (43.0%)	22 (36.7%)	21 (52.5%)	NS
moderate	39 (39.0%)	26 (43.3%)	13 (32.5%)	
severe	18 (18.0%)	12 (20.0%)	6 (15.0%)	

NS—‘not significant’.

**Table 6:** Distribution of patients in anxiety disorder severity groups (according to HADS-M), considering gender division.

Anxiety Disorder Severity Groups (According to HADS-M)	Total Group Size/ Frequency (n = 100)	Females (n = 60)	Males (n = 40)	*p*
no disorders	80 (80.0%)	41 (68.3%)	39 (97.5%)	0.001
borderline states	14 (14.0%)	15 (25.0%)	0 (0.0%)	
disorders	6 (6.0%)	4 (6.7%)	1 (2.5%)	

## Data Availability

The datasets generated and analyzed in the current study are available from the corresponding author upon reasonable request.
